# Genomic epidemiology, evolution, and transmission dynamics of porcine sapelovirus associated with diarrheic piglets

**DOI:** 10.1080/21505594.2026.2697085

**Published:** 2026-06-29

**Authors:** Xia Li, Xin‘ ao Ma, Xiaoyu Wang, Qingjun Li, Qi Yuan, Jiahua Wang, He Bai, Yong Liu, Feipeng Zhao

**Affiliations:** a School of Basic Medical Sciences, Mudanjiang Medical University, Mudanjiang, China; b School of Veterinary Medicine, Northwest A&F University, Yangling, China; c First Clinical Medical School, Mudanjiang Medical University, Mudanjiang, China; d College of Life Sciences, Mudanjiang Medical University, Mudanjiang, China

**Keywords:** Picornaviruses, porcine sapelovirus, epidemiology, evolution, transmission

## Abstract

Porcine sapelovirus (PSV) is increasingly detected in swine enteric disease complexes, but its evolutionary dynamics and transmission patterns remain insufficiently characterized. In this study, we analyzed 327 fecal samples collected from diarrheic piglets between 2015 and 2022 and identified persistent PSV detection in northeastern China. Because PSV was detected in the context of mixed enteric viral infections in this dataset, our data do not establish PSV as an independent causative agent of diarrhea. Comparative whole-genome and evolutionary analyses of globally circulating PSV strains revealed substantial genetic diversity, with higher apparent short-term substitution-rate estimates observed in the African and Japanese datasets. In China, inter-strain genetic recombination appeared to represent an additional driver of viral diversification. Temporal evolutionary analyses indicated a dynamic and complex evolutionary landscape within China. Phylogeographic reconstruction identified multiple putative transmission nodes within the currently available genome dataset, suggesting broad geographic dissemination of PSV lineages but not definitive source–sink relationships. These findings enhance our understanding of PSV genomic epidemiology and provide useful information for molecular surveillance of PSV and other diarrhea-associated viruses in swine populations.

## Introduction

Porcine sapelovirus (PSV) is classified within the genus Sapelovirus in the family Picornaviridae. According to the tenth report of the International Committee on Taxonomy of Viruses (ICTV), the genus Sapelovirus comprises two species, Sapelovirus A and Sapelovirus B, with Sapelovirus A represented by the single species PSV-1. Several unclassified sapeloviruses have also been isolated from diverse hosts, including bats, California sea lions, groundhogs, and mice. With advances in sequencing technologies, Boros et al. identified a potential novel PSV genotype in Hungary using viral metagenomics [1]. PSV is primarily transmitted via the fecal–oral route and has been associated with multisystemic clinical manifestations in swine, including diarrhea, pneumonia, encephalomyelitis, and reproductive disorders [2–9].

PSV viral particles are characterized by smooth surfaces, non-vesicular morphology, and nearly microspherical icosahedral structures with an approximate diameter of 30 nm [10,11]. Consistent with the composition of most viral particles, the PSV virion consists of a capsid and RNA, with a composition ratio 70% protein to 30% RNA [12]. The PSV genome is a single-stranded, positive-sense RNA molecule of approximately 7.5 kb, comprising a 5'-terminal VPg region, a 5' untranslated region (UTR), an intermediate coding region, a 3' UTR, and a 3'-terminal poly(A) tail [13]. The intermediate coding region contains a substantial open reading frame (ORF) that encodes a polyprotein precursor of approximately 2,330 amino acids. This polyprotein is subsequently cleaved by its own encoded protease into 12 mature functional proteins. Arranged sequentially from the 5' to the 3' end, these proteins include the leader peptide L, VP4, VP2, VP3, VP1, 2A, 2B, 2C, 3A, 3B, 3C, and 3D. Among these proteins, P1 (comprising VP4, VP2, VP3, and VP1) represents the viral structural proteins, while P2 (comprising 2A, 2B, and 2C) and P3 (comprising 3A, 3B, 3C, and 3D) represent the viral non-structural proteins [13]. Importantly, VP1 is situated on the external surface of the viral particle and is pivotal in eliciting the production of neutralizing antibodies. Furthermore, VP1 contains binding sites for cellular receptors, rendering it a crucial protein in the viral invasion of host cells [14].

Accumulating evidence indicates that PSV strains circulating in China share close genetic affinities with those reported globally. For instance, Zhang’s analysis revealed that PSV strains prevalent in Henan cluster tightly with strains from Zambia [14]. Similarly, Yang’s VP1-based phylogenetic assessment of PSV strains from Hunan demonstrated their close relatedness to counterparts from Germany, Spain, Japan, and Korea [15]. In a subsequent investigation, Yang further confirmed phylogenetic links between Hunan-derived strains and those circulating in the United States [16]. Collectively, these findings underscore the substantial genetic diversity and rapid evolutionary dynamics of PSV in China, suggesting that continued genomic surveillance is needed to monitor the emergence and spread of novel variants.

PSV infection frequently occurs alongside a spectrum of other viral pathogens—including Porcine Parvovirus (PPV), Porcine Epidemic Diarrhea Virus (PEDV), Classical Swine Fever Virus (CSFV), Porcine Reproductive and Respiratory Syndrome Virus (PRRSV), and Porcine Enterovirus (PEV)—resulting in complex co-infection landscapes that may complicate clinical interpretation and pathogen attribution [17,18]. Despite these notable epidemiological and clinical implications, current knowledge of PSV remains limited. These gaps highlight the need for systematic studies to elucidate the molecular epidemiology, evolutionary trajectories, and potential role of PSV in swine enteric disease complexes.

In this study, we identified persistent detection of Porcine Sapelovirus (PSV) in diarrheic piglet samples collected from northeastern China between 2015 and 2022. By integrating genomic, evolutionary, and phylogeographic analyses, we systematically characterized key aspects of PSV evolution and epidemiology, including co-infection patterns, genetic recombination, temporal signals, evolutionary rates, and inferred dispersal patterns. These analyses provide an improved understanding of PSV circulation dynamics and offer useful information for molecular surveillance and future epidemiological studies of PSV and other diarrhea-associated viruses in swine populations.

## Results

### PSV prevalence and sequencing in China

**Figure 1.**
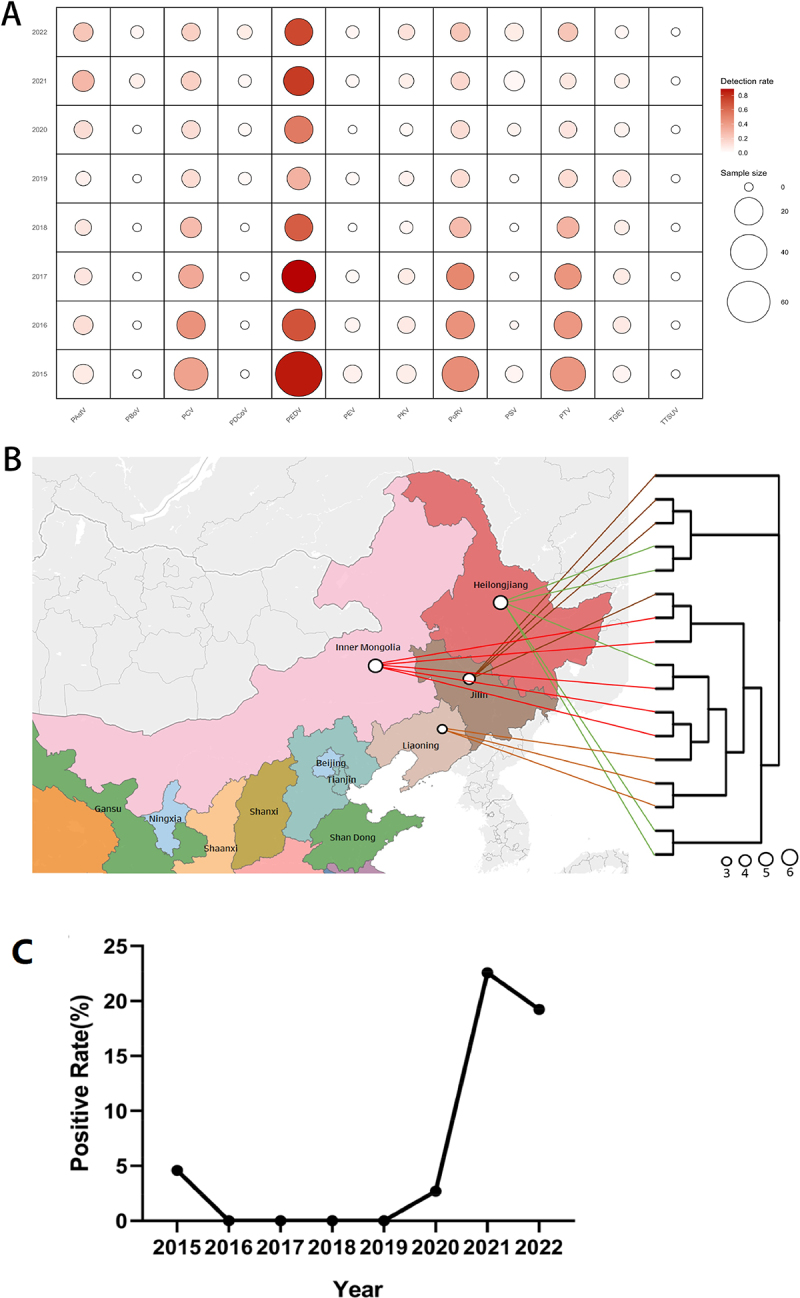
Characteristics of PSV detection in northeastern China from 2015 to 2022. (A) Schematic overview of 327 fecal samples collected from pigs between 2015 and 2022. The upset plot summarizes the twelve common swine diarrhea-associated viruses (PAstV, TBoV, PCV, PDCoV, PEDV, PEV, PKV, PoRV, PSV, PTV, TGEV, and TTSUV). Color shading and circle size indicate yearly sample numbers. (B) Geographic distribution of the 17 PSV whole-genome sequences obtained in this study from 2015 to 2022. The phylogenetic tree was constructed using the maximum-likelihood method with 1,000 bootstrap replicates. Circle sizes in each province denote the number of sequenced PSV genomes. (C) Annual PSV-positive rate from 2015 to 2022.

A total of 327 fecal samples were collected from piglets with diarrhea across four provinces or autonomous regions in northeastern China between 2015 and 2022 (Figure 1(A), Figure S1; Supplementary Table S1). The annual positive rates of the twelve diarrhea-associated viruses detected from 2015 to 2022 are shown in Figure S1. The clinical symptoms observed were consistent with those typically associated with enteric viral infections, including porcine sapelovirus (PSV), porcine epidemic diarrhea virus (PEDV), pseudorabies virus (PRV), and porcine deltacoronavirus (PDCoV) (Figure 1(A)). Although PEDV remains the predominant etiological agent of swine diarrhea, small RNA viruses such as PSV were persistently detected in diarrheic piglets over the surveillance period (Figure 1(C), Supplementary Table S3). The annual PSV-positive rate showed an increasing trend during the surveillance period from 2015 to 2022 (Figure 1(C)). This finding indicates that PSV has become an increasingly detectable enteric virus among diarrheic piglets in northeastern China, highlighting the need for continued molecular surveillance. Analysis of the full sample set showed that 66.89% of cases involved mixed viral infections. PSV was detected in 17 of the 327 fecal samples, corresponding to a sample-level positivity rate of 5.20% (Figure S2(A,B); Supplementary Table S3). At the farm level, PSV was detected in 12 of the 16 sampled farms, corresponding to a farm-level detection rate of 75.0%. The number of PSV-positive samples per positive farm ranged from 1 to 3 (Supplementary Table S8). Because farms were sampled after diarrheal disease was reported, this value should be interpreted as farm-level detection among affected farms rather than as population-level farm prevalence. Notably, all 17 PSV-positive samples subjected to complete genome sequencing were co-detected with other enteric viruses, including PEDV, PoRV, and PAstV. Therefore, these data indicate PSV co-circulation in diarrheic piglets but do not establish PSV as an independent cause of diarrhea. Complete PSV genomes were obtained from these 17 PSV-positive samples and were designated according to their sampling locations. Preliminary phylogenetic analysis classified these genomes into three major clades: one strain formed the first clade, twelve strains constituted the second clade, and the remaining four strains grouped into the third clade (Figure 1(B)).

### Analysis of PSV strain homologies, evolution, and recombination within and between regions

A comprehensive sequence analysis was conducted on 143 complete PSV genomes, including 17 newly sequenced genomes and 126 genomes retrieved from GenBank. Genome-wide diversity analysis showed that genetic variation was unevenly distributed across the PSV genome, with the VP1 region displaying the highest level of genetic diversity (Figure S3(A)). Because no phenotypic virulence assays, PSV-only infection group, or standardized clinical severity data were available, strains were not classified as virulent in this study. Therefore, subsequent analyses focused on genomic diversity, recombination, evolutionary rate, and phylogeographic patterns rather than direct virulence assessment.

**Figure 2.**
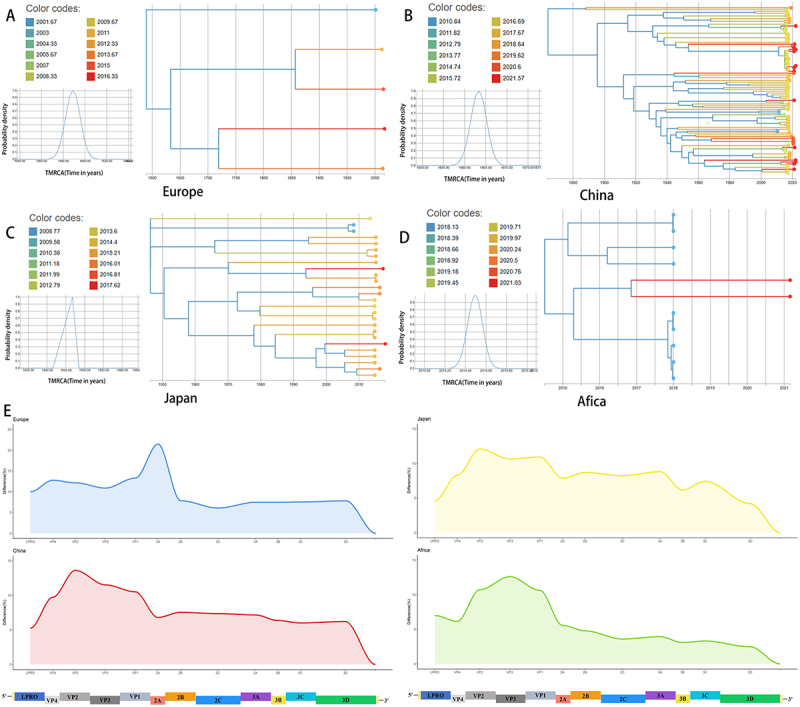
Evolution and recombination of PSV strains from four geographical regions: Europe, China, Japan, and Africa. (A–D) Maximum-likelihood time-scaled phylogenetic trees of complete PSV genomes from four geographical regions: (A) Europe, (B) China, (C) Japan, and (D) Africa. Different symbols indicate different sampling times. The x-axis represents time in years. Insets show the posterior probability density distributions of the time to the most recent common ancestor (TMRCA) for PSV strains from the four geographical regions. (E) Breakpoint distributions of recombination events in PSV strains from the four geographical regions.

To infer the common ancestors of PSV strains circulating in different geographic regions, we applied TreeTime for temporal phylogenetic reconstruction (Figure 2(A–D)). The most recent common ancestor (MRCA) of European PSV strains dates to the sixteenth century, whereas strains circulating in China trace back to the mid-nineteenth century. In contrast, the MRCA of Japanese strains emerged around 1945, and that of African strains around 2014. We further estimated the evolutionary rates of PSV strains across regions (Figure 2(A–D)). The global average nucleotide substitution rate was 5.489 × 10^−4^ substitutions/site/year. Regionally, the highest apparent nucleotide substitution rate was estimated for the African dataset (2.670 × 10^−2^ substitutions/site/year), followed by Japan (1.408 × 10^−2^) and China (1.666 × 10^−3^), whereas Europe showed the lowest estimate (5.181 × 10^−4^) (Figure S3(B–E); Supplementary Tables S5 and S6). However, these regional estimates should be interpreted cautiously because the datasets differed in sample size and temporal coverage, and the African sequences were limited in both number and temporal depth. Therefore, the elevated African rate is best interpreted as an apparent short-term rate estimate rather than definitive evidence that African PSV lineages intrinsically evolve faster than those from other regions. Temporal analyses revealed marked fluctuations in substitution rates between 2014 and 2021, with pronounced increases observed in 2014, 2017, and 2019 (Figure S3(F–M)). Spatial mapping of mutational patterns showed that strains circulating in China, Japan, and Africa accumulated mutations mainly within the VP4–VP1 region, while European strains displayed mutation hotspots in the 2A protein (Figure S4). Collectively, these findings suggest geographic heterogeneity in PSV evolutionary dynamics, with higher apparent short-term rate estimates observed in Africa and Japan; however, these differences may partly reflect uneven sampling intensity and temporal depth across regions. Notably, Chinese strains exhibited substitution rates above the global average, suggesting that continued surveillance is warranted to monitor potential changes in viral diversity, antigenicity, and regional dissemination.

The susceptibility of small RNA viruses to recombination prompted an analysis using RDP5 (version 5.05), which detected 180 recombination events among PSV strains (Figure S5(A)). Across all circulating strains, recombination breakpoints were primarily concentrated in the 2B and 3D proteins (Figure S5(B)). Regionally, recombination sites in China and Japan were likewise located in the 2B and 3D proteins, whereas African strains exhibited breakpoints predominantly within VP2 and VP3 (Figure 2(E)). Analysis of recombination frequency showed that Chinese PSV genomes harbored the highest number of recombination events, followed by those from Japan and Europe. Thirty events involved non-Chinese strains with recombination signals detected among Chinese PSV genomes, whereas sixteen events reflected the opposite pattern, with Chinese strains showing recombination signals among non-Chinese genomes. In total, 87 recombination events were identified among Chinese strains compared with 63 among strains from non-Chinese regions. Recombination events involving Chinese strains accounted for 48.3% of all global recombination events and 73.91% of those occurring within China. In contrast, strains circulating outside China contributed 35% of global recombination events and 70.59% of non-China events. These findings suggest that, within the currently available dataset, recombination signals were more frequently detected among Chinese PSV genomes. However, this pattern should be interpreted cautiously because regional sequence availability and sampling intensity are uneven. In addition, higher apparent short-term substitution-rate estimates were observed in the African and Japanese datasets, whereas recombination signals were more frequently detected in Chinese PSV genomes (Figure S3, Figure S5(B–E)).

### Haplotype dynamics of PSV

**Figure 3.**
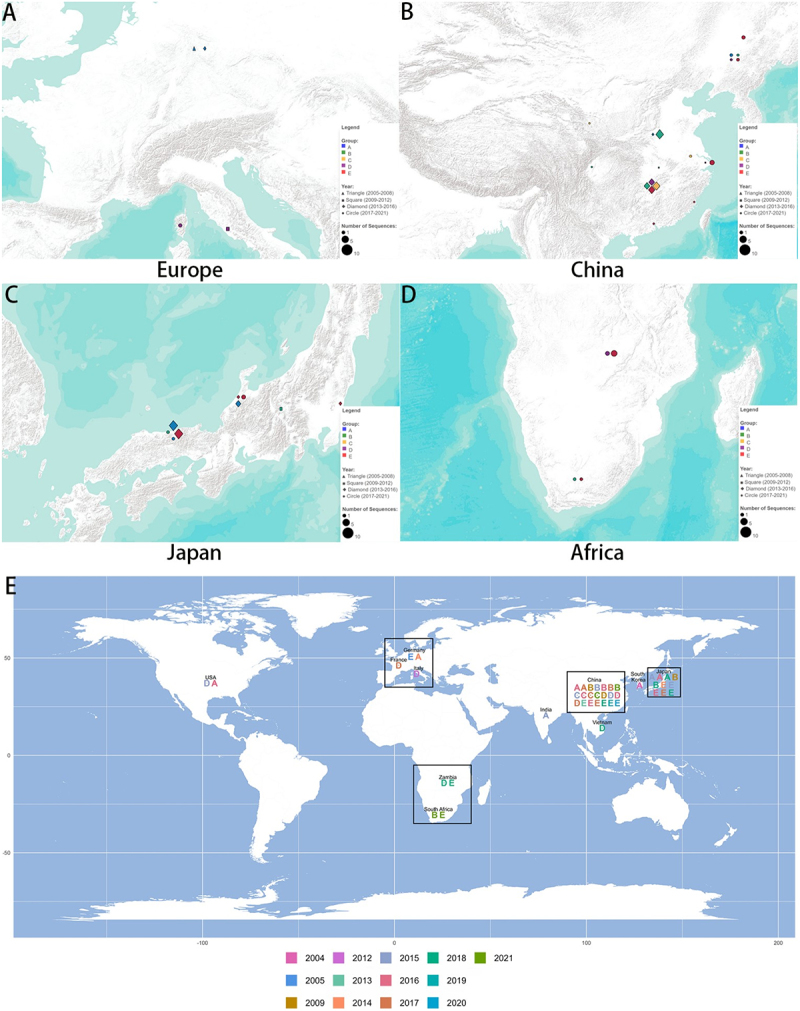
Haplotype dynamics of PSV. Five groups (A–E) of haplotypes were identified in Supplementary Figure. S6, Supplementary Material online. (A–D) 3C haplotype distribution in (A) Europe, (B) China, (C) Japan, and (D) South Africa. (E) Global distribution of various haplotype groups.

To clarify the temporal and genetic relationships underlying the evolution of PSV across different geographic regions, we performed a haplotype analysis based on the nucleotide sequences of the non-structural protein 3C (Figure S6). The 3C gene, a conserved component among small RNA viruses, plays a central role in viral genome replication. As shown in Figure 3 and Figure S6, PSV comprises 102 distinct haplotypes that cluster into five major haplotype groups. The geographic distribution of these haplotypes is outlined as follows: Chinese strains encompass five groups (A, B, C, D, and E). Among these, groups A, B, and E are also represented in Japanese strains. Strains from the United States fall within groups A and D, whereas strains from South Africa are distributed across groups B, D, and E. Notably, only group D haplotypes are detected in Italy and Vietnam, and France similarly harbors solely group D strains. In contrast, group A strains are prevalent in Korea, Germany, and India.

Regarding temporal patterns, the earliest reported detections of PSV haplotypes were as follows: Germany in 2005; Korea in 2004; China in 2009, 2013, 2015, and 2016; Japan in 2009, 2014, and 2015; India and the United States in 2015; Italy in 2012; France in 2017; Zambia and Vietnam in 2018; and South Africa, which reported notably later detections, in 2021. Collectively, these results suggest that, within the currently available dataset, haplotype diversification was more extensive among Chinese PSV strains than among strains from several other sampled regions, indicating a dynamic and regionally complex evolutionary landscape.

### Spatial and temporal reconstruction of the global spread of PSV

**Figure 4.**
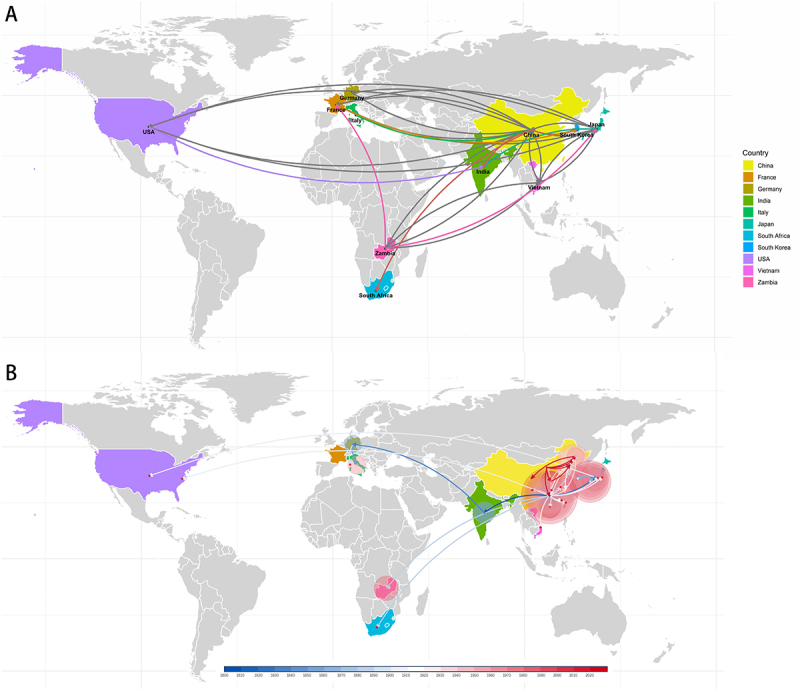
Spatiotemporal diffusion of PSV strains based on the currently available genome dataset.

To explore the putative global spatial and temporal transmission dynamics of PSV, we performed phylogeographic analyses using BEAST. Based on the currently available PSV genome dataset, the discrete phylogeographic model inferred Europe and Asia as well-connected regions in the global PSV transition network, with bidirectional transition signals between these regions (Figure 4(A)). Within Europe, France appeared as a highly connected node in the available dataset, with inferred viral transitions involving Germany, Japan, China, Zambia, and South Africa. In Asia, China was inferred as a highly connected node for viral lineage transitions in the current reconstruction, with model-supported links to Germany, France, Italy, Japan, Vietnam, Zambia, the United States, South Korea, India, and South Africa (Figure 4(A)). These inferred routes should be interpreted as model-supported transition signals conditional on the available sequence dataset rather than definitive evidence of direct import–export events.

(A) Discrete phylogeographic analysis was performed using Bayesian stochastic search variable selection (BSSVS). Inferred transition events are shown with 98% highest posterior density regions. Colors indicate inferred ancestral locations in the model reconstruction, while gray areas indicate model-supported bidirectional transition signals between sampled regions. (B) Continuous phylogeographic reconstruction was performed using a Bayesian relaxed random-walk model. The MCC tree and 98% highest posterior density regions were mapped based on trees subsampled from the post-burn-in posterior distribution. Nodes are scaled according to the reconstruction. The inferred spatial diffusion patterns should be interpreted as model-based signals conditional on the available PSV genome sequences.

The continuous phylogeographic model inferred an early spatial signal in Asia followed by subsequent expansion signals involving Europe, particularly Germany and France, within the limits of the available genomes (Figure 4(B)). Additional inferred expansion signals involved African and American regions, including Zambia and the United States, contributing to the reconstructed geographic distribution of sampled PSV lineages. However, because global PSV genomic sampling is incomplete and uneven, this pattern should not be interpreted as definitive evidence of the true geographic origin or complete migration history of PSV. Analyses of viral population dynamics over time revealed three periods of apparent global population expansion, occurring around 1850, 1916, and 2000, followed by sustained fluctuations at relatively high population levels (Figure 4(B)). Estimates of the effective population size for Chinese PSV lineages similarly indicated apparent expansions around 1916 and 2000, after which population size remained elevated (Figure S7(B)).

Discrete phylogeographic reconstruction within China suggested a predominantly north-to-south transition pattern among sampled Chinese PSV lineages, with Hunan Province appearing as a highly connected node in the inferred domestic transition network. Model-supported links were observed between Hunan and Jilin, Wuhan, Henan, Liaoning, and Guangdong. In total, Hunan showed inferred epidemiological links with 13 provinces, suggesting that it may represent an important sampled node in the domestic PSV transition network (Figure S7(A)). These findings were broadly consistent with patterns inferred from the continuous phylogeographic model (Figure S7(B)), but should be interpreted in the context of uneven provincial sampling.

In summary, these analyses provide a model-based reconstruction of PSV lineage movements using the currently available genome sequences and highlight the need for broader, more systematic genomic surveillance to refine global transmission inferences.

## Discussion

An epidemiological survey of 327 diarrheic piglet samples from northeastern China revealed the persistent circulation of small RNA viruses, including porcine sapelovirus (PSV), across Heilongjiang, Jilin, Liaoning, and Inner Mongolia during the surveillance period. The high frequency of mixed viral infections indicates that PSV circulates within complex enteric viral communities in diarrheic piglets. However, because PSV-only infections were not identified among the sequenced PSV-positive samples, this study cannot determine whether PSV independently causes diarrhea or primarily acts as a co-circulating virus in enteric disease complexes. The farm-level analysis further showed that PSV was detected in 12 of the 16 affected farms, indicating that PSV detection was not confined to a single farm. However, because the farms were selected by convenience sampling after diarrheal disease was reported, this farm-level detection rate should not be interpreted as the prevalence of PSV among all pig farms in northeastern China. The 17 PSV whole-genome sequences generated in this study enrich the available PSV genomic dataset and provide useful reference data for characterizing PSV genetic diversity in northeastern China. Comparative analyses of these genomes with globally sampled PSV strains suggested that Chinese strains in the available dataset exhibit greater genetic diversity than those found in Africa, Europe, or Japan. Moreover, higher apparent short-term substitution-rate estimates were observed in the African and Japanese datasets, whereas recombination signals were more frequently detected among Chinese PSV genomes in the available dataset. Although PSV was first identified in 1958 and subsequently reported in Australia in 1982 [19], the temporal and mechanistic drivers of recombination across different geographic regions have remained obscure. Our recombination analysis identified multiple recombination signals in the available global dataset, with a higher number of such signals detected among Chinese PSV genomes. Previous studies have shown that cross-border movements of live pigs can facilitate the spread of porcine epidemic diarrhea virus (PEDV) [20]. Our haplotype analysis is compatible with the possibility that animal movement and regional connectivity may contribute to PSV lineage distribution, although direct transmission routes cannot be established from these data. Specifically, the results suggest that Germany may represent an early sampled European reservoir or historical transmission node for PSV, whereas China appeared as a recombination-rich region in the currently available dataset. This pattern may be related to animal movement and live-pig trade, although direct transmission routes cannot be established from the available genomic data. These findings are consistent with haplotype distributions, in which Germany, Korea, and India harbor only a single haplotype, while Chinese strains showed the highest haplotype diversity within the currently available dataset, comprising up to five distinct groups.

The coexistence of an older inferred TMRCA for European lineages and a comparatively lower evolutionary rate suggests that PSV lineages in Europe may have reached a relatively stable, host-adapted evolutionary state after long-term circulation. This pattern is not necessarily contradictory: older endemic lineages may be subject to stronger purifying selection, transmission bottlenecks, and slower lineage turnover, resulting in a lower apparent substitution rate [21,22]. In contrast, the elevated evolutionary rate estimated for African PSV strains should be interpreted with particular caution. The African dataset was limited in size and temporal depth, with most available sequences derived from relatively recent sampling. Under such conditions, root-to-tip regression and molecular-clock analyses can be affected by short-term evolutionary rate inflation, in which transient mutations, incomplete purifying selection, founder effects, and narrow sampling windows produce higher apparent substitution-rate estimates [21,22]. Therefore, the high rate estimated for African strains should not be interpreted as definitive evidence of intrinsically faster PSV evolution in Africa. Instead, it likely reflects a combination of recent lineage sampling, limited temporal structure, and dataset composition. Similar caution is also warranted when comparing regional evolutionary rates across datasets with unequal sample sizes and sampling periods. The distinct regional mutation hotspots further support divergent evolutionary pressures. The accumulation of mutations in the VP4–VP1 region in China, Japan, and Africa may be associated with adaptation in capsid-exposed regions, especially because VP1 participates in receptor binding and is a major target of neutralizing antibodies [23,24]. Such mutations could therefore influence antigenicity, host-cell entry, immune recognition, and viral fitness. By contrast, the predominance of mutation hotspots in the European 2A region suggests that European PSV lineages may be shaped more by constraints on non-structural protein function, including viral polyprotein processing, replication efficiency, and host – virus interactions, rather than by rapid capsid antigenic diversification [25]. These divergent evolutionary routes imply that PSV surveillance should not rely exclusively on VP1-based genetic monitoring; instead, both structural and non-structural genomic regions should be incorporated to detect region-specific evolutionary signals with potential relevance to viral adaptation, antigenicity, and diagnostic sensitivity. Because the available PSV genomes are unevenly distributed across countries and sampling years, these regional differences should be interpreted as model-based evolutionary signals rather than definitive evidence of geographic origin or fixed evolutionary mechanisms. Nevertheless, these mechanistic interpretations require further validation using reverse genetics, viral replication assays, and serological cross-neutralization studies. This study provides a complementary framework for exploring the spatial and temporal relationships underlying PSV dissemination beyond conventional phylogenetic approaches. To our knowledge, this work represents the first haplotype-based investigation of PSV, and the five haplotype groups identified herein provide a valuable reference system for the rapid global classification of PSV strains.

We additionally conducted Bayesian discrete phylogeographic inference to explore putative spatial transition patterns of PSV based on the currently available genome dataset. Our results indicated that relatively independent lineages were present in the United States and South Africa, whereas China and several other sampled regions showed interconnected lineage distributions. The phylogeographic analyses further suggested that Germany may represent an early sampled European reservoir or historical transition node for PSV in the available dataset, while China and France appeared as highly connected nodes in the inferred transition network. However, these findings should be interpreted as conditional, model-based signals rather than definitive evidence that Germany was the true geographic origin of PSV, that China was the dominant global exporter, or that France was the principal European transmission hub. Because the global PSV genome dataset is derived largely from sequences available in GenBank rather than from systematic global surveillance, the inferred directionality of viral movement is sensitive to sampling density, temporal coverage, and uneven geographic representation. Many swine-producing regions remain unsampled or underrepresented for PSV genomes, which may bias ancestral-state reconstruction and source–sink inference. Therefore, the geographic origin, export routes, and regional transmission hubs inferred here should be viewed as dataset-dependent hypotheses requiring validation through broader and more balanced genomic surveillance.

In summary, this study improves our understanding of the transmission dynamics, evolutionary trajectories, and geographical distribution patterns of Porcine Sapelovirus (PSV) based on the currently available genome dataset. Our analyses suggest that PSV lineages sampled from Asia show substantial genetic diversity and recombination signals. Notably, five distinct viral haplotypes were identified in China, providing potentially valuable markers for PSV molecular surveillance and future epidemiological risk assessment. In contrast, strains sampled from India and Europe appeared relatively stable in the present dataset. France appeared as a well-connected European node in the phylogeographic reconstruction; however, this should not be interpreted as definitive evidence that France is the primary conduit for PSV dissemination in Europe. These findings support the need for strengthened international cooperation in animal genomic surveillance. These findings should be interpreted in light of several limitations. The field samples were collected mainly from diarrheic piglets in northeastern China, which may limit the generalizability of the epidemiological findings to other regions of China or to asymptomatic pigs. Moreover, no PSV-only infection group or asymptomatic control group was available, and the PSV-positive samples analyzed here were co-detected with other enteric viruses. Therefore, the present study cannot establish a causal relationship between PSV infection and diarrhea. In addition, although farm identifiers, sampling years, piglet ages, and clinical signs were recorded, detailed litter-level information and standardized diarrhea severity scores were not available for all samples, which may limit outbreak-level or litter-level epidemiological inference. The global PSV genome dataset also remains unevenly distributed across countries and sampling years, and many swine-producing countries currently lack publicly available PSV genome sequences. Because the global dataset was assembled largely from sequences available in GenBank rather than from systematic global surveillance, phylogeographic inference may be affected by sampling density, temporal coverage, and uneven geographic representation. These biases may influence estimates of viral origin, evolutionary rate, recombination frequency, source–sink directionality, and phylogeographic diffusion. This limitation is particularly relevant to the African dataset, for which the small number of available genomes and narrow temporal sampling window may inflate apparent substitution-rate estimates and reduce the robustness of direct regional comparisons. Therefore, the inferred geographic origins, transmission hubs, and regional evolutionary patterns should be interpreted cautiously as dataset-dependent hypotheses and require further validation through broader genomic surveillance and longitudinal sampling. Collectively, these findings provide a useful reference for molecular surveillance, epidemiological risk assessment, and future studies of PSV in swine enteric disease complexes.

## Conclusion

In conclusion, this study provides genomic and epidemiological evidence for the persistent detection and circulation of Porcine Sapelovirus (PSV) in diarrheic piglets from northeastern China. By employing an integrative analytic framework that combines genomic, evolutionary, and phylogeographic approaches, we provide a dataset-dependent reconstruction of PSV evolution and epidemiology, encompassing its co-infection context, recombination patterns, temporal signals, evolutionary tempo, and inferred regional dissemination patterns. These findings improve current knowledge of PSV ecology and evolutionary behavior and highlight the importance of broader, more balanced genomic surveillance to refine PSV transmission and evolutionary inferences in swine enteric disease complexes.

## Materials and methods

### Sample collection, identification, and sequencing

A total of 327 fecal samples were collected from diarrheic piglets in four provinces or autonomous regions of northeastern China, including Heilongjiang, Jilin, Liaoning, and Inner Mongolia, between 2015 and 2022 (Supplementary Table S1). The samples were obtained from 16 pig farms after diarrheal disease was reported in piglets, and the age of the sampled piglets ranged from 3 to 7 days. Available metadata, including sample ID, sampling year, province, farm identifier, piglet age, clinical signs, and diarrhea status, are provided in Supplementary Table S1. Diarrhea was clinically defined as the presence of loose, watery, or unformed feces, with or without dehydration, reduced appetite, vomiting, or growth retardation, as observed by farm veterinarians or animal caretakers. The farms were selected by convenience sampling rather than random sampling, and the samples represented individual diarrheic piglets from affected farms. Although farm identifiers and sampling years were recorded, detailed litter-level information was not available for all samples. Therefore, multiple samples from the same farm and year were considered farm-associated submissions rather than independent litter-level outbreak records. Verbal informed consent was obtained from all farm owners and animal keepers prior to sample collection, and participants were fully apprised of the purpose, procedures, volume, and intended use of the materials. Twelve viral pathogens associated with porcine diarrhea were detected using PCR or RT-PCR assays with virus-specific primers. The primer information is provided in Supplementary Table S2 and deposited in Mendeley Data. The raw pathogen-detection results for all 327 fecal samples are provided in Supplementary Table S3 and are available in the same dataset. Farm-level PSV detection was defined as the presence of at least one PSV-positive fecal sample from a sampled farm during the surveillance period. Farm-level units were defined by the combination of province and farm identifier. PSV-positive samples were subsequently subjected to metagenomic next-generation sequencing and whole-genome assembly as described below.

### Metagenomic sequencing, genome assembly, and quality control

PSV-positive fecal samples were subjected to metagenomic next-generation sequencing and whole-genome assembly by Comate Bioscience Co., Ltd. Briefly, total RNA was extracted from fecal suspensions and processed according to the company’s standard RNA virome sequencing workflow. The remaining RNA, including single-stranded and double-stranded RNA, was reverse-transcribed and converted into double-stranded cDNA. Qualified cDNA was randomly fragmented by ultrasonication and used for sequencing-library construction. Library preparation was performed according to the commercial service provider’s standard RNA virome protocol. The libraries were sequenced on an Illumina NovaSeq 6000/NovaSeq X Plus platform using a paired-end 150-bp sequencing strategy. Raw reads were quality-filtered using fastp to remove adapter sequences, low-quality reads, PCR duplicates, and polyX-containing reads. Host-derived and rRNA reads were removed by mapping clean reads against the Sus scrofa reference genome and the SILVA rRNA database using BWA v0.7.17. Alignments covering less than 80% of the read length were discarded. The remaining reads were assembled de novo using MEGAHIT v1.1.2 with the parameters --presets meta-large -- min-contig-len 300. Clean reads were mapped back to the assembled contigs using BWA v0.7.17 to evaluate read utilization and support contig assembly. Putative viral contigs were identified using CheckV based on HMM- and AAI-based approaches, with DIAMOND searches against viral reference databases, including checkv-db-v1.5, NCBI GenBank viral sequences, Riboviria, and ICTV-related viral databases. PSV-related contigs were further identified by sequence similarity searches against reference PSV genomes. Final assembled PSV genome sequences with complete coding regions and no internal ambiguous bases were retained for downstream analyses. The 17 newly assembled PSV genome sequences generated in this study were deposited in Mendeley Data as downloadable sequence files and are listed in Supplementary Table S4.

### Sequence analysis

Complete PSV genomes available in GenBank and sampled between 2001 and 2021 were retrieved from the NCBI GenBank database (https://www.ncbi.nlm.nih.gov/). This study compiled a total of 126 complete genome sequences of globally distributed PSV, in addition to 17 newly sequenced PSV genomes (Supplementary Tables S4 and S5). The alignment of all 143 sequences was performed using MEGA-X software (version 10.2.6).

### Phylogenetic and evolutionary dynamic analysis

Phylogenetic trees were constructed for each of the 143 genomes utilizing the maximum likelihood (ML) approach implemented in IQ-Tree version v1.6.12 software [26]. The ML tree was generated employing a General Time Reversible (GTR) model for nucleotide substitutions and Empirical Base Frequencies (F), Free Rate Model with 6 Categories (R6) model for rate heterogeneity, with 1,000 bootstrap replications. The genotype of each viral strain was determined by means of a phylogenetic tree. As outlined by Zhao et al. nucleotide sequence diversity for each specific locus was calculated [27]. To ensure accuracy and reliability, only sites containing a maximum of 10% ambiguous bases (N) or gaps (-), as identified by the sequencing process, were included in the calculation of nucleotide sequence diversity. The same parameters were used to assess similarity and divergence in the nucleotide sequences of the 3C proteins. The TreeTime software [28] was used to estimate apical distances, evolutionary rates, and time-proportional phylogenies of the viral strains. A coalescent-based nonparametric Skygrid prior was used in BEAST to estimate changes in effective population size over time [29]. Convergence was verified using the Tracer software, version 1.7 [30], with a burn-in period of 10% of the total chain length. The effective sample sizes for all parameters estimated using the BEAST software were greater than 200.

### Recombination analysis

In parallel, we also characterized the recombination patterns of PSV (Supplementary Table S7). The recombination analysis included strains from four geographic regions: China, Japan, Africa, and Europe. A phylogenetic network was constructed employing the NeighborNet method via the SplitsTree software [31]. Recombination events within the viral genome were identified using the Recombination Detection Program 5 (RDP5), as previously described [32]. Events were considered genuine recombination occurrences only if they satisfied the criteria established by at least three out of the seven implementations in RDP5, using a *p*-value threshold of 0.05. Following this, recombinant sequences were excluded, and the process was iteratively repeated until no additional recombination events were detected.

### Haplotype dynamic analysis

A total of 143 nucleic acid sequences of PSV nonstructural protein 3C were selected from the complete PSV genome. Haplotypes were inferred using DnaSP6 software [33]. Templeton, Crandall, and Sing (TCS) networks were constructed for all 3C haplotypes using POPART software [34,35] and subsequently adjusted manually using Cytoscape v3.8.2 [36].

### Time-calibrated phylogeny reconstruction and phylogeographic analysis of PSV

Bayesian evolutionary and phylogeographic analyses were conducted using the BEAST software, version 1.10.4, and the BEAGLE library, version 4.0.02.167 [37], with the objective of enhancing computational performance. The locus model employed in all BEAST analyses was GTR +F+G4 for two distinct codon partitions (1 + 2, 3). The Skygrid coalescent mode with uncorrelated relaxation clocks and strict molecular clocks was selected based on path and stepping stone sampling. Three distinct runs, each with a randomly generated seed, were conducted for a total of 800 million generations, yielding comparable results. The output was analyzed using Tracer v1.7.2 [30] to ensure that the effective sampling size (ESS) was greater than 200 for all parameters. In the phylogeographic analysis, the sampling countries were used as discrete features, with a total of 25 distinct discrete locations. The discrete phylogeographic analysis was conducted using a symmetric substitution model, and social networks were inferred through Bayesian Stochastic Search Variable Selection (BSSVS) [38]. The visualization of discrete propagation routes and the computation of the Bayes factor (BF) were performed using SpreaD3 v0.9.7 software [39]. The lognormal RRW model was selected for the continuous phylogeographic analysis [40], and the results were visualized using the R software packages ggplot2 v3.5.1, maps v3.4.2, and dplyr v1.1.4 [41].

## Supplementary Material

Supplementary_Tables_S1_S8_PSV_epidemiology.xlsx

## Data Availability

The complete genome sequences of all reference PSV strains analyzed in this study are available from the National Center for Biotechnology Information (NCBI), and their accession numbers are listed in Supplementary Table S5. The datasets generated and analyzed in this study, including the 17 newly assembled PSV genome sequences, sample information for the 327 fecal samples, primer sequences used for PCR/RT-PCR detection of the 12 viral pathogens, raw pathogen-detection results for the 327 fecal samples, farm-level PSV detection summary, estimated evolutionary rates, and recombination-event data, are available in Mendeley Data: zhao, feipeng (2026), “Genomic Epidemiology, Evolution, and Transmission Dynamics of Porcine Sapelovirus Associated with Diarrheic Piglets V2,” Mendeley Data, V3, doi: 10.17632/8mmzn7jgp2.3, under a Creative Commons Attribution 4.0 International (CC BY 4.0) license. The 17 newly assembled PSV genome sequences are provided as downloadable sequence files within this dataset.
